# Valosin‐containing protein counteracts ATP‐driven dissolution of FUS condensates through its ATPase activity *in vitro*

**DOI:** 10.1002/1873-3468.70328

**Published:** 2026-03-16

**Authors:** Hitomi Kimura, Shin‐ichi Tate, Kyota Yasuda

**Affiliations:** ^1^ Graduate School of Integrated Sciences for Life Hiroshima University Japan; ^2^ WPI‐SKCM Hiroshima University Japan; ^3^ Research Center for the Mathematics on Chromatin Live Dynamics (RcMcD) Hiroshima University Japan; ^4^ Institute of Advanced Mathematical Sciences (MIMS) Meiji University Tokyo Japan; ^5^ Graduate School of Medical Life Science Yokohama City University Japan

**Keywords:** adenosine triphosphate, amyotrophic lateral sclerosis, fused in sarcoma, liquid–liquid phase separation, valosin‐containing protein

## Abstract

Fused in sarcoma (FUS) forms phase‐separated condensates implicated in amyotrophic lateral sclerosis (ALS). Although millimolar ATP concentrations paradoxically dissolve FUS condensates through hydrotropic activity, condensates nevertheless persist in cells, suggesting active regulatory mechanisms. Here, using a reconstituted system, we show that the AAA+ATPase valosin‐containing protein (VCP) counteracts ATP‐driven dissolution of FUS condensates. VCP preserved both wild‐type and ALS‐linked P525L condensates under high ATP conditions, and this protection required catalytic ATPase activity rather than stable partitioning into condensates. The effect was abolished by the D2‐specific inhibitor ML240. Our findings establish direct biochemical evidence that VCP ATPase activity maintains FUS condensates under high ATP conditions, highlighting ATPase‐driven enzymatic control of liquid–liquid phase separation as a potential general principle with implications for neurodegeneration.

## Graphical abstract

Biomolecular condensates formed by fused in sarcoma (FUS) are dissolved by high ATP concentrations yet persist in cells. Using a reconstituted system, we demonstrate that valosin‐containing protein (VCP), an AAA+ ATPase, counteracts ATP‐driven dissolution of FUS condensates through its D2 ATPase activity. Surprisingly, VCP provides stronger protection despite lower ATP consumption than apyrase, a nonspecific nucleotidase, suggesting mechanisms beyond bulk ATP depletion.

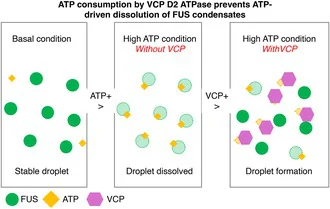

## Abbreviations

**ALS**, amyotrophic lateral sclerosis

**ATP**, adenosine triphosphate

**DMSO**, dimethyl sulfoxide

**DTT**, dithiothreitol

**FTLD**, frontotemporal lobar degeneration

**FUS**, Fused in sarcoma

**GFP**, green fluorescent protein

**IPTG**, isopropyl‐β‐D‐thiogalactopyranoside

**LCD**, low‐complexity domain

**LLPS**, liquid–liquid phase separation

**MBP**, maltose‐binding protein

**NLS**, nuclear localization signal

**PEG**, polyethylene glycol

**RFP**, red fluorescent protein

**SEM**, standard error of the mean

**VCP**, valosin‐containing protein

**WT**, wild‐type

Biomolecular condensates formed through liquid–liquid phase separation (LLPS) have emerged as a fundamental organizing principle in cell biology [1, 2]. These membraneless organelles dynamically compartmentalize biochemical reactions and are implicated in numerous cellular processes, from RNA metabolism to signal transduction. Dysregulation of LLPS is increasingly recognized as a common feature of neurodegenerative diseases, where RNA‐binding proteins aberrantly aggregate into pathological inclusions [1, 2].

The aberrant aggregation of proteins is a common pathological hallmark of numerous neurodegenerative diseases, including amyotrophic lateral sclerosis (ALS) and frontotemporal lobar degeneration (FTLD) [3]. A central protein implicated in these conditions is the DNA/RNA‐binding protein, Fused in Sarcoma (FUS) [4, 5]. FUS contains an N‐terminal low‐complexity domain (LCD) that drives its phase separation [6, 7] and is typically localized to the nucleus where it participates in diverse biological processes, such as transcription, RNA splicing, and DNA repair [5]. Disease‐associated mutations, particularly the severe P525L variant located in the nuclear localization signal (NLS), disrupt nuclear import and lead to cytoplasmic mislocalization and inclusion formation [6, 8, 9, 10]. These mutations accelerate the transition of FUS from dynamic, liquid‐like condensates to pathological, solid‐like aggregates [8, 11]. Although FUS granulation has physiological roles, its conversion into excessive and irreversible aggregates is thought to be a primary driver of neuronal cell death [10, 11, 12, 13].

The regulation of FUS condensate formation and dissolution is critically dependent on ATP, the cell's energy currency. ATP acts as a biological hydrotrope, exerting a biphasic effect on FUS phase separation depending on its concentration [14, 15, 16, 17, 18, 19, 20]. Whereas low concentrations of ATP facilitate FUS LLPS, the high physiological concentrations found in the cytoplasm (> 4.0 mm) conversely act to dissolve FUS condensates [14, 15, 16, 17, 18, 19, 20]. This presents a central paradox: How do FUS condensates persist in an ATP‐saturated cellular environment? This implies active mechanisms to locally modulate ATP levels.

A prime candidate for this regulatory mechanism is the multifunctional AAA+ ATPase, valosin‐containing protein (VCP, also known as p97). VCP belongs to the AAA+ (ATPases Associated with diverse cellular Activities) family and contains two distinct ATPase domains (D1 and D2) arranged in a double‐ring hexameric structure [21]. VCP is a critical component of the cellular proteostasis network, and notably, mutations in VCP are also a direct cause of familial ALS [22], placing FUS and VCP at a critical nexus of the disease. Our previous work identified VCP as a key factor in this mechanism. We demonstrated that VCP is a component of FUS condensates and that its ATPase activity regulates their residence time in cells by consuming intragranular ATP [23]. Based on this discovery, we proposed a model in which VCP functions as a ‘imer’ for FUS condensate turnover. In this model, VCP plays a dual role: it first stabilizes newly formed condensates by consuming ATP from high cytosolic levels, but its prolonged activity may lead to ATP depletion, which in turn destabilizes the condensates and targets them for clearance.

While our previous *in vivo* studies suggested that VCP regulates FUS condensate stability through ATP consumption [23], this model was based on indirect evidence from cellular experiments where multiple factors could contribute to the observed effects. Direct biochemical evidence demonstrating that VCP ATPase activity alone is sufficient to modulate FUS condensate stability was lacking. The present study aimed to test this hypothesis using a minimal reconstituted system containing only purified FUS, VCP, and ATP, thereby establishing whether VCP's effect on FUS condensates is an intrinsic biochemical property independent of cellular cofactors. Here, we show that VCP does not directly affect the physical properties of FUS condensates but potently reverses their dissolution under high ATP concentrations. This restoration of FUS condensates is entirely dependent on VCP's enzymatic function, as it is completely blocked by the specific ATPase inhibitor ML240. These findings provide direct biochemical evidence that VCP ATPase activity regulates FUS condensate stability.

## Materials and methods

### Plasmids

For plasmids encoding pMAL‐His‐MBP‐TEV‐GFP‐FUS, we amplified the GFP‐conjugated mouse FUS coding sequence, carrying a homologous mutation to the human P525L mutant used in our previous works [23, 24, 25]. Then, it was cloned into pMAL‐C6T (N0378S; NEB) with NotI and BamHI. pET28a/His‐TEV‐RFP was constructed by inserting the RFP coding sequence to pET28a plasmid with NdeI and BamHI. We ordered the plasmid pET‐6xHis‐TEV‐hVCP‐mScarlet3 from the vendor (vector ID: VB241016‐1188qvr; Vector builder).

### Purification of MBP‐GFP‐FUS

The plasmid encoding MBP‐GFP‐FUS (WT or P525L) was transformed into E. coli BL21 (DE3) (DS250; Biodynamic Laboratory Inc., Bunkyo‐ku, Tokyo, Japan). Cultures were grown in LB medium with 50 μg·mL^−1^ Carbenicillin at 37 °C to an OD600 of 0.6, then induced with 1 mm isopropyl‐β‐D‐thiogalactopyranoside (IPTG; MS Technosystems, GEN‐S‐02122, Amagasaki, Hyogo, Japan) and incubated at 16 °C for 18–20 h. Cells were harvested and resuspended in Buffer A (20 mm Tris–HCl, pH 7.4, 200 mm NaCl, 1 mm dithiothreitol (DTT)), and 10% Glycerol. The cell suspension was subjected to sonication and clarified by centrifugation. The supernatant was loaded onto an amylose resin (E8021L; New England Biolabs, Ipswich, MA, USA) and washed with Buffer A and eluted with Buffer A added with 10 mm maltose. Proteins were dialyzed against buffer solution (50 mm Tris–HCl, pH 7.0, 2 m Urea) for 17 h at 4 °C. The dialyzed protein was applied to a cation exchanger (HiTrap SP FF, 17‐5157‐01; Cytiva, Marlborough, MA, USA) and purified by a linear salt gradient from 0 M to 1.5 m KCl in Buffer B. The fractions containing the target protein were collected and subjected to dialysis against the buffer solution (100 mm Tris–HCl, pH 7.4, 140 mm KCl) at 4 °C for 16 h. The dialyzed protein solution was filtered with a sterilizing device having a 0.2 μm pore size (New Stella disk, S‐1302; KURABO, Osaka, Japan) to remove the protein aggregates before storage at 4 °C before use.

### Purification of RFP

The plasmid encoding RFP was transformed into E. coli BL21 (DE3). Cultures were grown in LB medium with 50 μg·mL^−1^ kanamycin at 37 °C to an OD600 of 0.6 and then induced with 0.5 mm IPTG and incubated at 16 °C for 18–20 h. Cells were harvested and resuspended in Buffer C (50 mm Tris–HCl, pH 9.0, 200 mm NaCl, 2 m urea, and 20 mm imidazole), lysed by sonication, and clarified by centrifugation. The supernatant was loaded onto a Ni column (HiTrapTM FF, cat no.: 17525501; Cytiva) and washed with buffers C, D (4 m NaCl), and E (50 mm NaCl), and eluted with buffer F (500 mm imidazole). Protein was dialyzed against the buffer solution (100 mm Tris–HCl, pH 7.4, and 140 mm KCl). The dialyzed protein solution was filtered with a sterilizing device having a 0.2 μm pore size to remove the protein aggregates before storage at 4 °C before use.

### Purification of hVCP‐mScarlet3

The plasmid encoding hVCP‐mScarlet3 was transformed into E. coli BL21 (DE3). Cultures were grown in LB medium with 50 μg·mL^−1^ Carbenicillin at 37 °C to an OD600 of 0.6, then induced with 0.4 mm IPTG, and incubated at 16 °C for 18–20 h. Cells were harvested and resuspended in buffer G (50 mm Tris–HCl, pH 7.0, 0.1 mm DTT). The cell suspension was subjected to sonication and clarified by centrifugation. The supernatant was transferred to a beaker and made up to 100 mL with buffer G. The solution was placed on ice, and 40% ammonium sulfate was added while stirring. The solution was stirred for 1 h on ice, and the mixture was centrifuged. The supernatant was loaded onto a Ni column and washed with the buffer solution (50 mm Tris–HCl, pH 7.0, 0.1 mm DTT, 100 mm NaCl, 20 mm Imidazole) and eluted with the buffer solution (50 mm Tris–HCl, pH 7.0, 0.1 mm DTT, 100 mm NaCl, 500 mm Imidazole). Protein was dialyzed against the buffer solution (100 mm Tris–HCl, pH 7.4, and 140 mm KCl). The dialyzed protein solution was filtered with a sterilizing device having a 0.2 μm pore size to remove the protein aggregates before storage at 4 °C before use.

### FUS condensate formation

To prevent aggregation during concentration, 200 mm arginine hydrochloride (L‐(+)‐Arginine Monohydrochloride, 03323‐45; Nacalai Tesque, Kyoto, Japan) was added to MBP‐GFP‐FUS. Purified proteins were concentrated using 10 kDa molecular weight cutoff devices (Amicon Ultra Centrifugal Filter concentrator, UFC901024; Merck, Burlington, MA, USA), stored on ice, and equilibrated to room temperature for 30 min before use. FUS condensates were formed by mixing MBP‐GFP‐FUS (20 μm final), RNA (200 ng·μL^−1^), arginine hydrochloride (100 mm), and PEG 3350 (10%; 202 444‐250G; Sigma‐Aldrich, St. Louis, MO, USA), followed by a 30‐min incubation at room temperature.

### *In vitro* FUS condensate assays

FUS condensates were formed as described above. For all assays, samples were equilibrated for 5 min before initial imaging (0 min), with subsequent imaging at 60 min.

Basal conditions (Fig. 1): RFP or hVCP‐mScarlet3 (0, 30, or 60 μm) was added and incubated for 25 min.

**Fig. 1:**
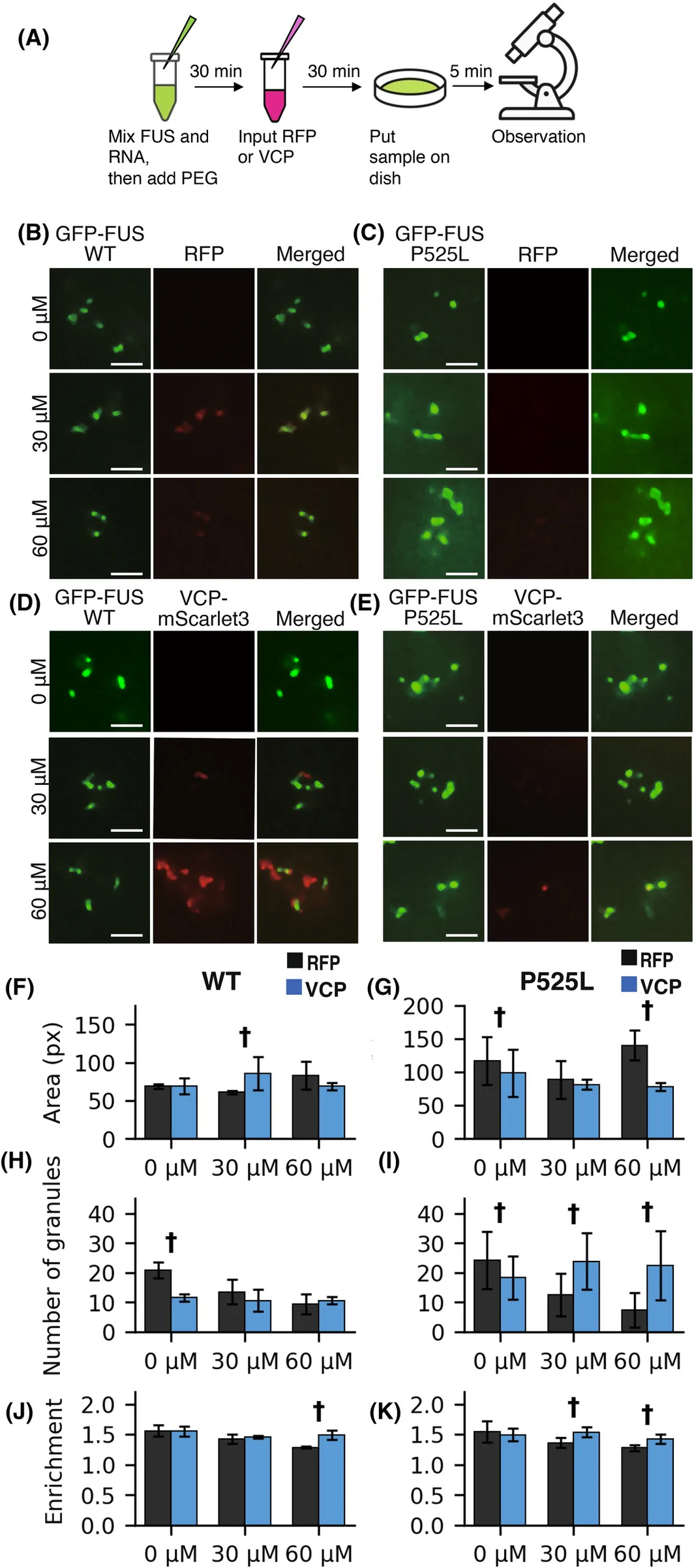
Valosin‐containing protein (VCP) does not alter fused in sarcoma (FUS) condensate formation under basal conditions. (A) Schematic of the experimental procedure. (B, C) Representative images of green fluorescent protein (GFP)‐FUS [wild‐type (WT) and P525L] with 0, 30, and 60 μm red fluorescent protein (RFP). (D, E) Representative images of GFP‐FUS with 0, 30, and 60 μM hVCP‐mScarlet3. VCP shows diffuse localization. (F–K) Quantification of condensate area (F, G), number (H, I), and enrichment (J, K) for WT and P525L FUS. No significant difference was observed between RFP and VCP conditions. Scale bars: 5 μm. Data represent mean ± SEM from *n* = 3 independent experiments (10 images per experiment). †indicates large effect size (|Cliff's δ| ≥ 0.474). Statistical details for all comparisons are provided in Table S1.

High ATP conditions (Fig. 2): ATP (0, 2.5, 5, or 12.5 mm) was added for 30 min, followed by RFP or hVCP‐mScarlet3 (60 μm).

**Fig. 2:**
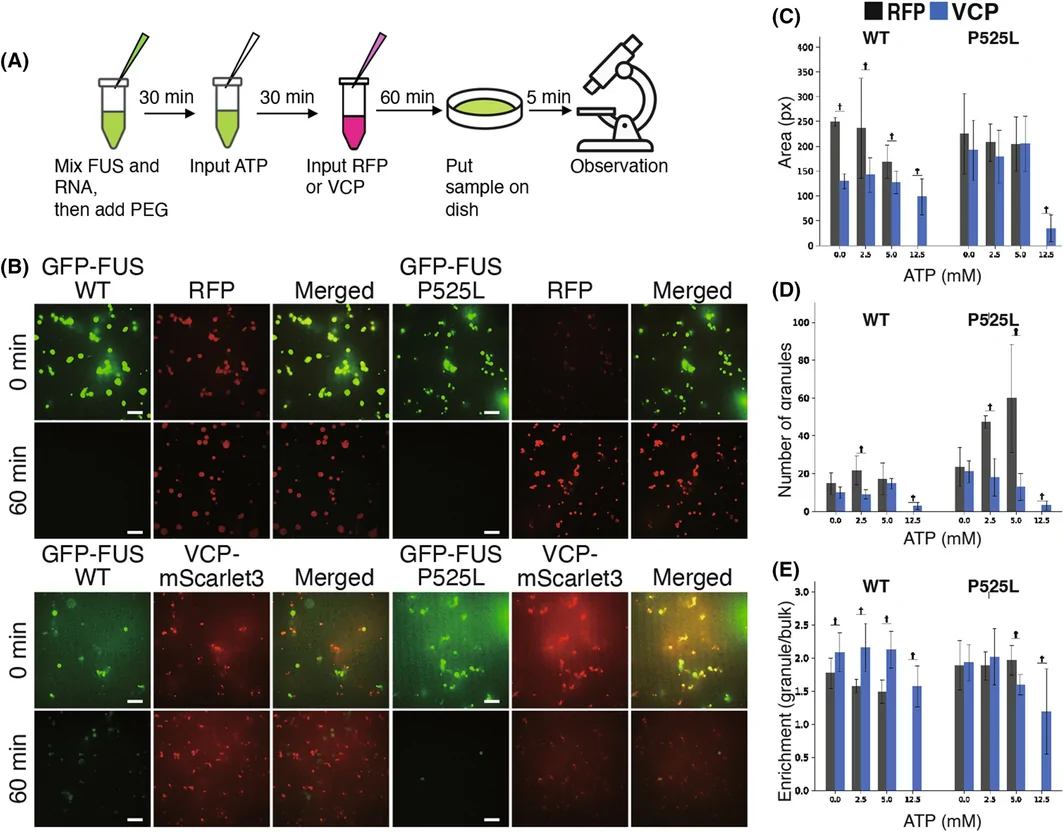
Valosin‐containing protein (VCP) prevents fused in sarcoma (FUS) condensate dissolution in high ATP. (A) Schematic of the high‐ATP assay. ATP is added after condensate formation, followed by VCP or red fluorescent protein (RFP). (B) Representative images showing that 12.5 mm ATP dissolves FUS condensates in the presence of RFP (60 min) but not VCP. Condensates are largely dissolved with RFP but preserved with VCP at 60 min. (C‐E) Quantification of mean condensate area (C), number of condensates (D), and enrichment (E) at various ATP concentrations (0, 2.5, 5.0, 12.5 mm). Scale bars: 5 μm. Data represent mean ± SEM from *n* = 3 independent experiments (10 images per experiment). †indicates large effect size (|Cliff's δ| ≥ 0.474). Statistical analysis (Mann–Whitney U‐test and Cliff's delta) is summarized in Table S1.

RFP‐LLPS control (Fig. S1): RFP (60 μm), BSA (20 μm), RNA (200 ng·μL^−1^), arginine hydrochloride (100 mm), and PEG 3350 (polyethylene glycol 3350 Da; 10%) were mixed. ATP (12.5 mm) was added for 25 min.

ATPase inhibitors (Fig. 3 and Fig. S2): RFP or hVCP‐mScarlet3 was preincubated with DMSO, ML240 (SML1071‐5MG; Sigma‐Aldrich), CB‐5083 (S8101; Selleck Chemicals), or NMS‐873 (S7285; Selleck Chemicals, Houston, TX, USA) for 30 min before addition (60 μm) to ATP‐treated condensates.

**Fig. 3:**
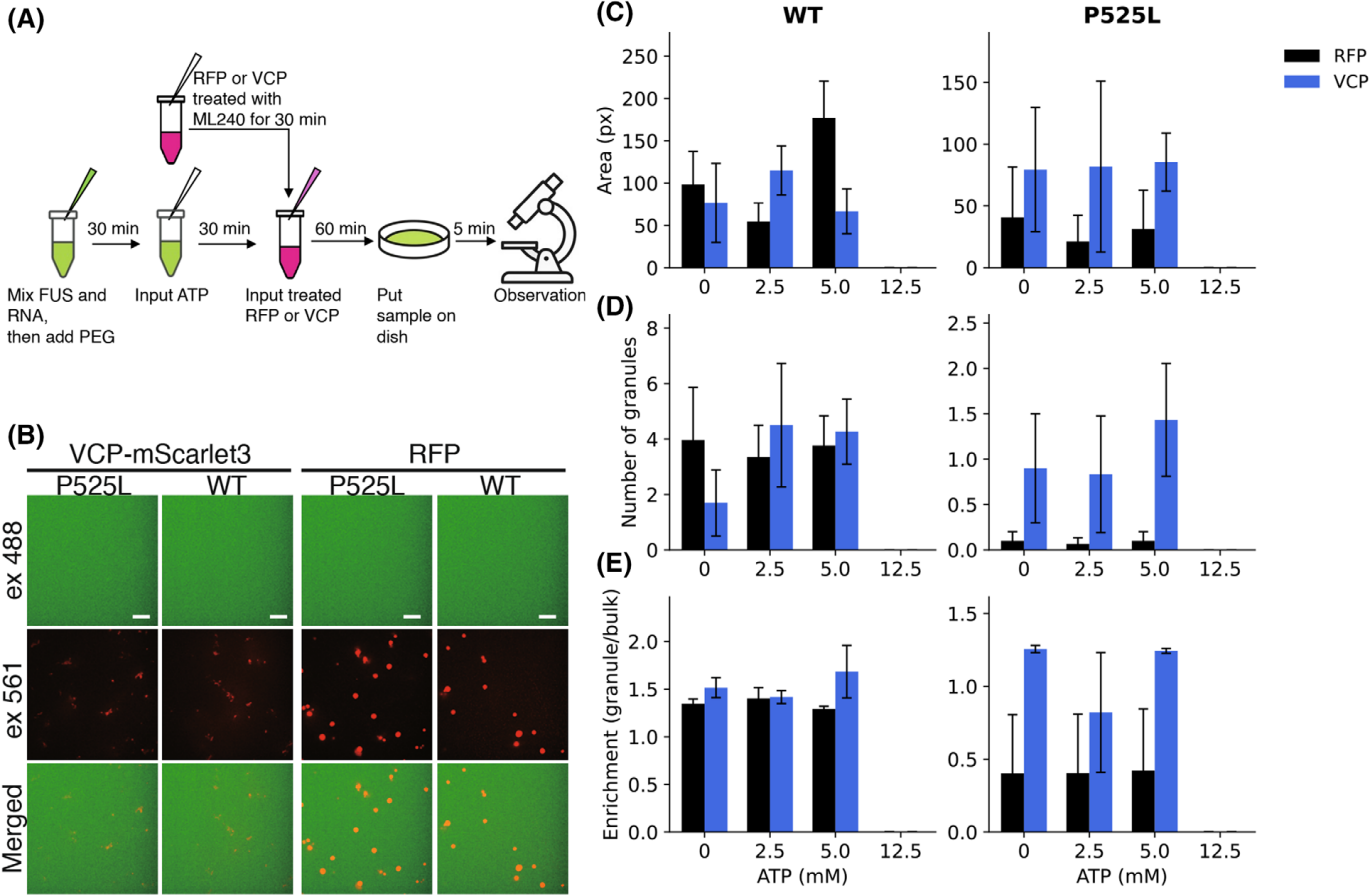
D2 ATPase activity is essential for valosin‐containing protein (VCP)‐mediated condensate regulation. (A) Schematic of the inhibitor assay. (B) Representative images of fused in sarcoma (FUS) condensates with VCP in the presence of ML240 at 12.5 mm ATP (60 min). ML240 treatment leads to condensate dissolution at high ATP. (C–E) Quantification of condensate area (C), enrichment (D), and number (E) comparing wild‐type (WT) and P525L FUS under inhibitor treatments. Scale bars: 10 μm. Data represent mean ± SEM from *n* = 3 independent experiments (10 images per experiment). †large effect size (|Cliff's δ| ≥ 0.474; Mann–Whitney U‐test). Statistical details are provided in Table S1.

Apyrase (Fig. 4): ATP (12.5 mm) was added for 30 min, then RFP (60 μm) or apyrase (M0398S; 1 or 5 U·mL^−1^; NEB) with 8% DMSO. Heat‐inactivated apyrase (100 °C, 10 min; 5 U·mL^−1^) served as a control.

**Fig. 4:**
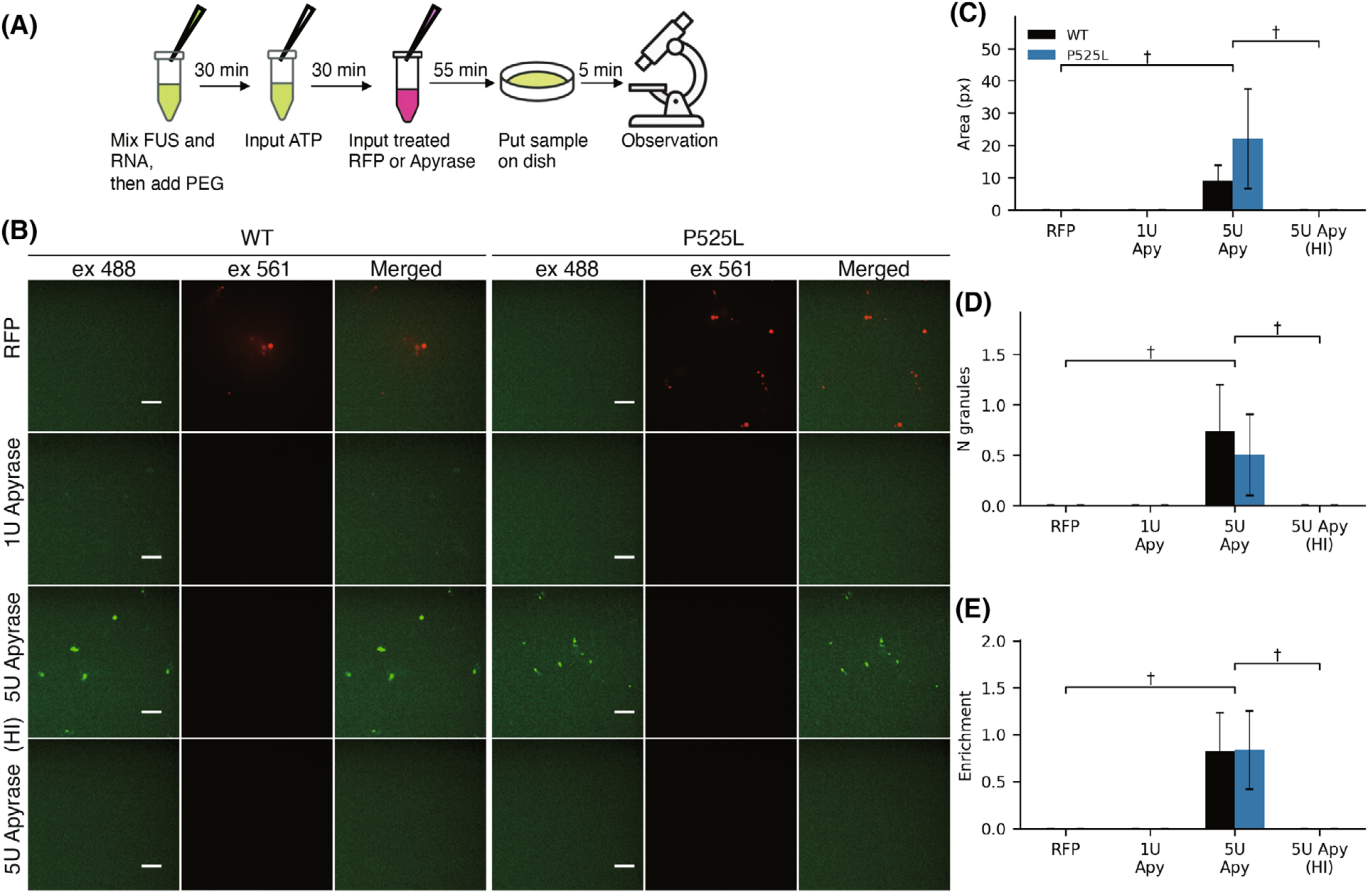
Apyrase recapitulates valosin‐containing protein (VCP)‐mediated condensate protection. (A) Schematic of the apyrase assay. Fused in sarcoma (FUS) condensates are formed, exposed to 12.5 mm ATP, then treated with red fluorescent protein (RFP), apyrase (1 or 5 U·mL^−1^), or heat‐inactivated (HI) apyrase. (B) Representative images of green fluorescent protein (GFP)‐FUS [wild‐type (WT) and P525L] under each condition at 60 min. Scale bars: 10 μm. (C–E) Quantification of condensate area (C), number (D), and enrichment (E). Data represent mean ± SEM from *n* = 3 independent experiments (10 images per experiment). * *P* < 0.05, †indicates large effect size (|Cliff's δ| ≥ 0.474; Mann–Whitney U‐test). Statistical details are provided in Table S1.

### ATP measurement assay

To assess VCP ATPase activity, ATP levels were measured using CellTiter‐Glo 2.0 Cell Viability Assay (G9242; Promega, Madison, WI, USA). FUS condensates were formed as described above. After adding 12.5 mm ATP for 30 min, RFP, hVCP‐mScarlet3, or apyrase was added. RFP or hVCP‐mScarlet3 (60 μm final) was preincubated with 8% DMSO or ATPase inhibitors (ML240, CB‐5083, or NMS‐873; 60 μm) for 30 min before addition. Apyrase (5 U·mL^−1^ final) was preincubated with 8% DMSO for 30 min. After 60 min of incubation at room temperature, an equal volume of CellTiter‐Glo 2.0 reagent was added and incubated for 10 min. Samples were mixed by pipetting, centrifuged briefly, and the supernatant was transferred to a 96‐well plate. Luminescence was measured after 20 min using a plate reader (TriStar LB 941; Berthold Technologies, Bad Wildbad, Germany). Three independent experiments were performed.

### Image acquisition and analysis

Microscopic images were acquired using an inverted microscope (IX83; Olympus, Tokyo, Japan) equipped with a spinning‐disk confocal unit (CSU‐W1; YOKOGAWA, Tokyo, Japan). Images were collected as Z‐stacks (0.2 μm step size, 21 slices) using a 100× oil‐immersion objective lens (NA 1.4, UPLSAPO 100XO; Olympus). GFP was excited using a 488‐nm laser, and RFP or mScarlet3 using a 561‐nm laser (OBIS; Coherent Inc., Santa Clara, CA, USA). Z‐stacks were converted to maximum‐intensity projections and analyzed using Fiji (imagej, NIH) [26] and custom python scripts for batch quantification. All images within each experiment were acquired using identical settings (laser power, exposure time, and gain).

Condensates were segmented using local adaptive thresholding (block size = 51 pixels, offset = 0). Objects were filtered by minimum area (15 pixels) and circularity (≥ 0.3) to exclude noise and nonspherical artifacts. These parameters were empirically optimized to minimize false‐positive detection while retaining visually identifiable condensates. The same parameters were applied consistently across all experimental conditions.

### Statistical analysis

Statistical comparisons were performed using the Mann–Whitney U‐test (two‐sided) to account for non‐normal distributions in condensate parameters. Effect sizes were calculated using Cliff's delta (δ) to assess the magnitude of differences independent of sample size. A *P*‐value < 0.05 was considered statistically significant. All detailed statistical results, including n numbers, *P*‐values, and effect sizes for every comparison, are provided in Table S1.

## Results

### VCP does not alter FUS condensate stability under basal conditions

To test whether VCP functions as a structural component or regulator of FUS condensates, we reconstituted FUS LLPS *in vitro*. We examined both wild‐type (WT) FUS and the ALS‐linked P525L mutant to determine whether disease‐associated mutations affect VCP‐mediated regulation.

Both WT and ALS‐linked mutant (P525L) FUS formed spherical condensates in the presence of RNA (Fig. 1A–C). The addition of VCP (0–60 μm) did not alter the number, size, or enrichment of these condensates compared with the RFP control across a range of concentrations (Fig. 1F–I). Importantly, VCP did not significantly alter condensate properties compared with RFP controls in either WT or P525L FUS, and no significant differences were observed between the two genotypes (Table S1). Furthermore, VCP did not strongly colocalize with FUS condensates (Enrichment = 1.4–1.5; Fig. 1D,E,J,K), values close to 1.0 (no enrichment) and well below the > 2.0 typically observed for stably incorporated condensate components. These results indicate that VCP does not function as a structural regulator of FUS condensates under basal conditions, regardless of disease‐associated mutations.

### VCP counteracts FUS condensate dissolution induced by high ATP

ATP is known to dissolve FUS condensates at high concentrations. We tested whether VCP could counteract this effect. In our assay, FUS condensates were preformed, exposed to ATP, and then treated with VCP or RFP (Fig. 2A). In the presence of 12.5 mm ATP, FUS condensates in the RFP control samples were largely dissolved after 60 min (Fig. 2B–D; RFP condition). In contrast, the addition of VCP prevented this dissolution, significantly attenuating dissolution and preserving condensate number and area at levels comparable to low ATP conditions (Fig. 2B–D; VCP condition). Notably, at the 0‐min time point, condensates were present in all conditions. Despite variability in condensate size distributions (Table S1), the protective effect of VCP was consistent and reproducible. Under high ATP conditions (12.5 mm), VCP treatment consistently preserved a visible population of FUS condensates, whereas condensates were extensively dissolved and often completely absent in the RFP controls (Fig. 2B). This protective effect was observed for both FUS‐WT and FUS‐P525L (Fig. 2C–E). At 12.5 mm ATP, condensates were absent in all RFP‐treated replicates (0/3 trials for both WT and P525L) but consistently present with VCP treatment (3/3 trials for WT, 2/3 for P525L). Effect sizes were large (Cliff's δ approached −1.0 for WT), reflecting near‐complete separation between conditions. Statistical details for all comparisons are provided in Table S1. Both WT and P525L FUS condensates showed largely comparable sensitivity to ATP‐driven dissolution and similar protection by VCP, with no significant differences between genotypes (Table S1). This mutation‐independent protection suggests that VCP's effect is intrinsic to its ATPase activity rather than dependent on specific interactions with FUS variants.

### VCP D2 ATPase activity is required for FUS condensate maintenance

To investigate the underlying molecular mechanism, we examined the effects of ATPase inhibitors targeting distinct domains of VCP. We utilized ML240, a selective inhibitor of the VCP D2 ATPase domain, and CB‐5083, an ATP‐competitive inhibitor that targets both D1 and D2 domains (Fig. 3A) [27].

Treatment with ML240 completely abolished the protective effect of VCP under high ATP conditions (Fig. 3B–E and Table S1). In the presence of ML240 (60 μm, 30 min preincubation), FUS condensates dissolved to a similar extent as observed in the RFP control, indicating a complete loss of VCP‐mediated condensate maintenance. Under ML240 treatment, both WT and P525L condensates dissolved at high ATP, though P525L showed a trend toward greater sensitivity to dissolution (Table S1). This observation is consistent with previous reports that P525L accelerates the liquid‐to‐solid transition and may reflect altered condensate material properties.

We also tested CB‐5083, an ATP‐competitive inhibitor that targets the nucleotide‐binding pocket of both D1 and D2 domains. Unexpectedly, CB‐5083 (60 μm, 30 min preincubation) did not abolish VCP's protective effect under the same conditions (Fig. S2A–D; Table S1). Both WT and P525L condensates remained protected by VCP despite CB‐5083 treatment. To address this, we tested NMS‐873, an allosteric D2 inhibitor with a distinct mechanism from ML240 [28]. NMS‐873 also abolished VCP's protective effect (Fig. S2E–H), consistent with ML240 results. The differential effects of CB‐5083 versus ML240/NMS‐873 may reflect distinct binding modes under high ATP conditions. To directly assess ATP consumption, we measured ATP levels using a luminescence assay (Fig. S3). VCP reduced ATP by 27% (*P* < 0.001), and this consumption was significantly inhibited by ML240 (*P* = 0.025) but not by CB‐5083 or NMS‐873. The correlation between bulk ATP consumption and condensate protection was consistent for ML240, whereas the lack of detectable ATP reduction by NMS‐873 in this assay, despite its effect on condensate protection, may reflect differences in assay sensitivity or local versus bulk ATP consumption.

### Reducing ATP concentration is sufficient for condensate maintenance

To determine whether VCP's protective effect is specific or whether any ATP‐hydrolyzing enzyme could similarly preserve FUS condensates, we tested apyrase, a nonspecific nucleotidase. Preformed FUS condensates were exposed to 12.5 mm ATP and then treated with apyrase at different concentrations (Fig. 4A).

Treatment with 5 U·mL^−1^ apyrase partially preserved FUS condensates under high ATP conditions, with detectable condensates observed in both WT and P525L samples at 60 min (Fig. 4B–E). In contrast, lower concentration (1 U·mL^−1^) and heat‐inactivated apyrase failed to protect condensates, which dissolved similarly to RFP controls. These results confirm that enzymatic ATP hydrolysis is sufficient for condensate maintenance, though the effect of apyrase appeared less robust than the VCP‐mediated protection observed in parallel experiments (Fig. 2). Both WT and P525L showed similar responses to apyrase treatment (Table S1), consistent with the mutation‐independent protection observed with VCP.

## Discussion

Our *in vitro* data support a role for VCP as a regulator of FUS LLPS stability, specifically by counteracting the hydrotropic effect of high ATP. The fact that ML240 (D2 inhibitor) blocked this effect while VCP itself did not strongly partition into the condensates suggests a catalytic mechanism: VCP likely operates at the condensate interface or in the surrounding microenvironment [29], consuming ATP and thereby reducing the concentration below the threshold required for dissolution. The hydrotropic effect of ATP on phase separation remains an area of active investigation [14, 30], with recent studies suggesting that the mechanism may involve both direct protein interactions and bulk solvent effects. This supports our ‘ATPase timer’ hypothesis [23]. Although variability was observed, the protective effect of VCP was reproducible across independent experiments.

Similar ATPase‐driven quality control mechanisms have been observed in other contexts, such as the disassembly of stress condensates (SGs) by VCP in mammalian cells [31], where VCP also facilitates autophagosomal clearance of protein aggregates [32], and the regulation of phase‐separated condensates in *C. elegans* [33]. Although some quantitative parameters did not consistently reach statistical significance, likely reflecting inherent heterogeneity in condensate size and number in this reconstituted system, the reproducible and condition‐dependent persistence of FUS condensates in the presence of VCP under high ATP conditions supports the presence of a robust ATPase‐dependent effect.

The differential effects of VCP inhibitors provide mechanistic insight. Both ML240 (ATP‐competitive D2 inhibitor) and NMS‐873 (allosteric D2 inhibitor) abolished VCP's protective effect on FUS condensates, while CB‐5083 (D1/D2 ATP‐competitive inhibitor) did not. Direct measurement of ATP consumption supported the imaging results for ML240: VCP reduced ATP levels by 27%, and this activity was significantly inhibited by ML240, whereas CB‐5083 and NMS‐873 showed no significant effect in this bulk assay (Fig. S3).

The failure of CB‐5083 to inhibit VCP function in our system likely reflects competition with the high ATP concentrations (12.5 mm) used in our assay. As an ATP‐competitive inhibitor, CB‐5083 must compete with excess ATP for binding to the nucleotide pocket, reducing its effective inhibition under these conditions. In contrast, the allosteric inhibitors ML240 and NMS‐873 bind to sites distinct from the ATP‐binding pocket, enabling effective inhibition regardless of ATP concentration. This observation has practical implications: allosteric targeting of the D2 domain may be more effective for modulating VCP activity in ATP‐rich environments, such as the cytoplasm.

A limitation of our pharmacological approach is that we cannot definitively exclude a contribution from the D1 ATPase domain, as D1‐specific inhibitors are not currently available. All characterized VCP inhibitors target either the D2 domain specifically (ML240, NMS‐873) or both domains (CB‐5083). Future studies using Walker B mutants (e.g., D1‐E305Q, D2‐E578Q, or double mutants) will be necessary to dissect the individual contributions of each ATPase domain to condensate regulation. However, structural and biochemical studies indicate that the D2 domain is the primary driver of ATP hydrolysis and substrate processing in VCP [34], with the D1 domain functioning primarily in hexamer stabilization and nucleotide sensing, supporting our interpretation that D2 activity is central to the observed effects.

Structural studies indicate that the D2 domain is the primary driver of VCP's substrate translocation and unfolding activity [34]. ML240 inhibited both bulk ATP consumption and condensate protection. NMS‐873 abolished condensate protection but did not measurably reduce bulk ATP levels in this assay, suggesting that it may act through local ATP consumption at condensate interfaces or affect VCP conformational dynamics rather than bulk hydrolysis. CB‐5083 has entered clinical trials for cancer therapy [35], demonstrating that modulating VCP activity is therapeutically viable.

A limitation of the current study is that we have not tested whether other physiologically relevant ATPases (e.g., Hsp70‐based chaperone systems or RNA helicases) can similarly counteract ATP‐driven dissolution of FUS condensates. While our apyrase experiments demonstrate that ATP hydrolysis *per se* is sufficient, VCP's unique structural features—including its hexameric ring architecture and high processivity—may enable efficient local ATP consumption at condensate interfaces that differ from nonspecific nucleotidases. Future studies comparing VCP with other cellular ATPases will be necessary to fully establish the specificity of this regulatory mechanism.

Our apyrase experiments (Fig. 4) demonstrate that ATP hydrolysis *per se* is sufficient for condensate maintenance, suggesting that VCP's protective effect operates primarily through ATP consumption rather than through specific protein–protein interactions with FUS.

Interestingly, quantitative comparison of VCP and apyrase revealed that ATP consumption efficiency does not directly correlate with condensate protection. Apyrase depleted > 99% of ATP yet preserved fewer condensates (N granules: 0.73 vs 2.87; Area: 9.07 vs 98.41 px) compared with VCP, which consumed only 27% of ATP. This disproportionality suggests that VCP may exert additional effects beyond bulk ATP depletion. One possibility is that VCP's hexameric architecture enables sustained local ATP consumption at condensate interfaces, creating microenvironments with reduced ATP concentrations that favor condensate stability. Alternatively, VCP may transiently interact with condensate components or modulate condensate material properties through mechanisms partially independent of bulk ATP hydrolysis. Distinguishing between these possibilities will require future studies with VCP mutants and direct visualization of VCP‐condensate interactions.

Although VCP hydrolyzes ATP to ADP, whereas apyrase generates AMP and inorganic phosphate, both enzymes reduce ATP concentration, which appears to be the critical parameter for condensate maintenance. Nevertheless, VCP may be uniquely suited for this role in cells due to its hexameric architecture, processivity, and localization to stress granules. Unlike apyrase, VCP is subject to cellular regulation through cofactors, such as UFD1‐NPL4 and p47, enabling spatiotemporal control of ATP consumption at specific condensates.

Regarding control conditions, Fig. S1 shows that RFP alone can also form condensates with limited ATP sensitivity in our simplified *in vitro* system [36, 37]. This behavior deviates from some established observations of FUS LLPS, in which ATP acts as a strong modulator of condensate stability [14, 15, 16, 17, 18, 19, 20]. Importantly, however, this does not affect the interpretation of VCP‐dependent regulation, as the ATP‐protective effect was specific to FUS condensates and was not observed in RFP controls. Thus, our core finding demonstrates that VCP specifically counteracts the ATP‐driven dissolution of FUS condensates, rather than exerting a nonspecific effect on protein assemblies.

Our previous in‐cell work suggested that the ALS‐linked P525L mutant interacts less efficiently with VCP, leading to altered condensate turnover [23]. In contrast, the present *in vitro* reconstitution shows that VCP preserves both WT and P525L condensates to a similar extent. This apparent discrepancy likely reflects the simplified nature of our system, which lacks cellular cofactors, such as transport receptors or UFD1–NPL4 that modulate VCP recruitment [38, 39, 40, 41]. Nevertheless, this mutation‐independent protection suggests that the biophysical mechanism is intrinsic to VCP ATPase activity and that VCP may similarly regulate other ALS‐associated condensates. Indeed, VCP suppresses TDP‐43 toxicity in fly models [42], and whether it modulates FUS pathology *in vivo* remains to be tested.

Our findings have implications for both normal physiology and disease. Under physiological conditions, FUS forms dynamic condensates that participate in transcription, RNA splicing, and DNA damage repair [5]. These condensates must be tightly regulated to prevent aberrant accumulation. VCP is recruited to stress granules and other RNA–protein condensates during cellular stress [29, 31], where it facilitates their disassembly through ubiquitin‐dependent pathways. Our data suggest an additional, perhaps earlier role: VCP may initially stabilize nascent condensates by locally consuming ATP, preventing premature dissolution in the ATP‐rich cytoplasm. This ‘timer’ function could allow condensates to mature and perform their physiological functions before VCP‐mediated clearance is initiated.

In the context of ALS pathophysiology, both VCP and FUS mutations cause familial disease [4, 22], placing them at a critical nexus. VCP mutations (e.g., R155H and R191Q) impair its ATPase activity and substrate processing, which could disrupt the normal regulation of FUS condensates. Indeed, we previously showed that VCP regulates FUS granule residence time in cells [23]. The P525L FUS mutation, while showing similar VCP‐mediated protection in our reconstituted system, exhibits altered condensate dynamics in cells due to cytoplasmic mislocalization and loss of nuclear import receptor interactions [8, 9, 10]. Understanding how disease mutations affect VCP‐FUS interplay may reveal therapeutic opportunities for ALS and related disorders.

Regarding VCP recruitment to condensates, while our enrichment data show that VCP does not stably partition into FUS condensates (enrichment ~ 1.4–1.5), this does not exclude transient or peripheral interactions. VCP may act at condensate interfaces rather than within the dense phase, consistent with its established role in extracting ubiquitinated substrates from various cellular structures [38]. Local ATP consumption at these interfaces could create microenvironments favoring condensate stability, which would be consistent with the disproportionate effects we observed comparing VCP and apyrase.

Our findings may extend beyond FUS to other ALS‐linked RNA‐binding proteins. TDP‐43, like FUS, undergoes LLPS and forms pathological inclusions in ALS. Given that VCP mutations cause familial ALS [22] and VCP plays a central role in cellular proteostasis networks [38], VCP may serve as a common regulator of multiple ALS‐associated protein condensates. Understanding how VCP modulates the material properties and turnover of these condensates could provide insights into disease mechanisms and therapeutic strategies.

Together, our findings reveal that VCP ATPase activity can counteract ATP‐driven dissolution of biomolecular condensates. This enzymatic control of LLPS may represent a general regulatory principle with implications for cellular organization and the pathogenesis of ALS and related neurodegenerative diseases.

## Author contributions

KY and S‐iT. were involved in the conceptualization. HK performed all the experiments. KY performed all the data analysis. KY was involved in the writing of the manuscript. S‐iT was involved in the supervision. KY and S‐iT were involved in the project administration.

## Supporting information


**Fig. S1.** Control assays for LLPS components.
**Fig. S2**. CB‐5083 and NMS‐873 differentially affect VCP‐mediated condensate protection.
**Fig. S3**. Direct measurement of ATP consumption by VCP.

**Table S1.** Statistical analysis for all figures.

## Data Availability

The data supporting the findings of this study are available from the corresponding author (KY: kyotay12@hiroshima-u.ac.jp) upon request.

## References

[1] Banani SF , Lee HO , Hyman AA and Rosen MK (2017) Biomolecular condensates: organizers of cellular biochemistry. Nat Rev Mol Cell Biol 18, 285–298. 10.1038/nrm.2017.7 PMC7434221 28225081

[2] Shin Y , Berry J , Pannucci N , Haataja MP , Toettcher JE and Brangwynne CP (2017) Spatiotemporal control of intracellular phase transitions using light‐activated optoDroplets. Cell 168, 159–171. 10.1016/j.cell.2016.11.054 PMC5562165 28041848

[3] Neumann M , Sampathu DM , Kwong LK , Truax AC , Micsenyi MC , Chou TT , Bruce J , Schuck T , Grossman M , Clark CM *et al*. (2006) Ubiquitinated TDP‐43 in frontotemporal lobar degeneration and amyotrophic lateral sclerosis. Science 314, 130–133. 10.1126/science.1134108 17023659

[4] Kwiatkowski TJ Jr , Bosco DA , Leclerc AL , Tamrazian E , Vanderburg CR , Russ C , Davis A , Gilchrist J , Kasarskis EJ , Munsat T *et al*. (2009) Mutations in the FUS/TLS gene on chromosome 16 cause familial amyotrophic lateral sclerosis. Science 323, 1205–1208. 10.1126/science.1166066 19251627

[5] Lagier‐Tourenne C , Polymenidou M , Hutt KR , Vu AQ , Baughn M , Huelga SC , Clutario KM , Ling SC , Liang TY , Mazur C *et al*. (2012) Divergent roles of ALS‐linked proteins FUS/TLS and TDP‐43 intersect in processing long pre‐mRNAs. Nat Neurosci 15, 1488–1497. 10.1038/nn.3230 PMC3586380 23023293

[6] Molliex A , Temirov J , Lee J , Coughlin M , Kanagaraj AP , Kim HJ , Mittag T and Taylor JP (2015) Phase separation by low complexity domains promotes stress granule assembly and drives pathological fibrillization. Cell 163, 123–133. 10.1016/j.cell.2015.09.015 PMC5149108 26406374

[7] Burke KA , Janke AM , Rhine CL and Fawzi NL (2015) Residue‐by‐residue view of in vitro FUS granules that bind the C‐terminal domain of RNA polymerase II. Mol Cell 60, 231–241. 10.1016/j.molcel.2015.09.006 PMC4609301 26455390

[8] Patel A , Lee HO , Jawerth L , Maharana S , Jahnel M , Hein MY , Stoynov S , Mahamid J , Saha S , Franzmann TM *et al*. (2015) A liquid‐to‐solid phase transition of the ALS protein FUS accelerated by disease mutation. Cell 162, 1066–1077. 10.1016/j.cell.2015.07.047 26317470

[9] Vance C , Rogelj B , Hortobagyi T , De Vos KJ , Nishimura AL , Sreedharan J , Hu X , Smith B , Ruddy D , Wright P *et al*. (2009) Mutations in FUS, an RNA processing protein, cause familial amyotrophic lateral sclerosis type 6. Science 323, 1208–1211. 10.1126/science.1165942 PMC4516382 19251628

[10] Dormann D , Rodde R , Edbauer D , Bentmann E , Fischer I , Hruscha A , Than ME , Mackenzie IR , Capell A , Schmid B *et al*. (2010) ALS‐associated fused in sarcoma (FUS) mutations disrupt Transportin‐mediated nuclear import. EMBO J 29, 2841–2857. 10.1038/emboj.2010.143 PMC2924641 20606625

[11] Murakami T , Qamar S , Julie G , Rees E , Miyashita A , Ana R , Fiona C , Kronenberg‐Versteeg D , Li Y , Yang S‐P *et al*. (2015) ALS/FTD mutation‐induced phase transition of FUS liquid droplets and reversible hydrogels into irreversible hydrogels impairs RNP granule function. Neuron 88, 678–690. 10.1016/j.neuron.2015.10.030 PMC4660210 26526393

[12] Nomura T , Watanabe S , Kaneko K , Yamanaka K , Nukina N and Furukawa Y (2014) Intranuclear aggregation of mutant FUS/TLS as a molecular Pathomechanism of amyotrophic lateral sclerosis. J Biol Chem 289, 1192–1202. 10.1074/jbc.M113.516492 PMC3887186 24280224

[13] Hofweber M , Hutten S , Bourgeois B , Spreitzer E , Niedner‐Boblenz A , Schifferer M , Ruepp MD , Simons M , Niessing D , Madl T *et al*. (2018) Phase separation of FUS is suppressed by its nuclear import receptor and arginine methylation. Cell 173, 706–719. 10.1016/j.cell.2018.03.004 29677514

[14] Patel A , Malinovska L , Saha S , Wang J , Alberti S , Krishnan Y and Hyman AA (2017) ATP as a biological hydrotrope. Science 356, 753–756. 10.1126/science.aaf6846 28522535

[15] Aida H , Shigeta Y and Harada R (2022) The role of ATP in solubilizing RNA‐binding protein fused in sarcoma. Proteins 90, 1606–1612. 10.1002/prot.26335 35297101

[16] He Y , Kang J , Lim L and Song J (2020) ATP binds nucleic‐acid‐binding domains beyond RRM fold. Biochem Biophys Res Commun 522, 826–831. 10.1016/j.bbrc.2019.11.180 31791586

[17] Kang J , Lim L , Lu Y and Song J (2019) A unified mechanism for LLPS of ALS/FTLD‐causing FUS as well as its modulation by ATP and oligonucleic acids. PLoS Biol 17, e3000327. 10.1371/journal.pbio.3000327 PMC6590835 31188823

[18] Kang J , Lim L and Song J (2018) ATP enhances at low concentrations but dissolves at high concentrations liquid‐liquid phase separation (LLPS) of ALS/FTD‐causing FUS. Biochem Biophys Res Commun 504, 545–551. 10.1016/j.bbrc.2018.09.014 30205960

[19] Kang J , Lim L and Song J (2019) ATP binds and inhibits the neurodegeneration‐associated fibrillization of the FUS RRM domain. Commun Biol 2, 223. 10.1038/s42003-019-0463-x PMC6586847 31240261

[20] Kitamura K , Tsukui I , Sasaki F , Shiramasa Y , Arayama M , Morishita M , Oshima A , Kitazawa S , Kameda T and Kitahara R (2025) ATP as a key modulator of fused‐in‐sarcoma phase separation and aggregation: insights into amyotrophic lateral sclerosis pathogenesis. J Mol Biol 437, 169295. 10.1016/j.jmb.2025.169295 40516596

[21] Zhang X , Shaw A , Bates PA , Newman RH , Gowen B , Orlova E , Gorman MA , Kondo H , Dokurno P , Lally J *et al*. (2000) Structure of the AAA ATPase p97. Mol Cell 6, 1473–1484. 10.1016/s1097-2765(00)00143-x 11163219

[22] Johnson JO , Mandrioli J , Benatar M , Abramzon Y , Van Deerlin VM , Trojanowski JQ , Gibbs JR , Brunetti M , Gronka S , Wuu J *et al*. (2010) Exome sequencing reveals VCP mutations as a cause of familial ALS. Neuron 68, 857–864. 10.1016/j.neuron.2010.11.036 PMC3032425 21145000

[23] Yasuda K , Watanabe TM , Kang MG , Seo JK , Rhee HW and Tate SI (2022) Valosin‐containing protein regulates the stability of fused in sarcoma granules in cells by changing ATP concentrations. FEBS Lett 596, 1412–1423. 10.1002/1873-3468.14353 35445401

[24] Yasuda K , Zhang H , Loiselle D , Haystead T , Macara IG and Mili S (2013) The RNA‐binding protein Fus directs translation of localized mRNAs in APC‐RNP granules. J Cell Biol 203, 737–746. 10.1083/jcb.201306058 PMC3857475 24297750

[25] Yasuda K , Clatterbuck‐Soper SF , Jackrel ME , Shorter J and Mili S (2017) FUS inclusions disrupt RNA localization by sequestering kinesin‐1 and inhibiting microtubule detyrosination. J Cell Biol 216, 1015–1034. 10.1083/jcb.201608022 PMC5379945 28298410

[26] Schindelin J , Arganda‐Carreras I , Frise E , Kaynig V , Longair M , Pietzsch T , Preibisch S , Rueden C , Saalfeld S , Schmid B *et al*. (2012) Fiji: an open‐source platform for biological‐image analysis. Nat Methods 9, 676–682. 10.1038/nmeth.2019 PMC3855844 22743772

[27] Chou TF , Li K , Frankowski KJ , Schoenen FJ and Deshaies RJ (2013) Structure‐activity relationship study reveals ML240 and ML241 as potent and selective inhibitors of p97 ATPase. ChemMedChem 8, 297–312. 10.1002/cmdc.201200520 PMC3662613 23316025

[28] Magnaghi P , D'Alessio R , Valsasina B , Avanzi N , Rizzi S , Asa D , Gasparri F , Cozzi L , Cucchi U , Orrenius C *et al*. (2013) Covalent and allosteric inhibitors of the ATPase VCP/p97 induce cancer cell death. Nat Chem Biol 9, 548–556. 10.1038/nchembio.1313 23892893

[29] Markmiller S , Soltanieh S , Server KL , Mak R , Jin W , Fang MY , Luo EC , Krach F , Yang D , Sen A *et al*. (2018) Context‐dependent and disease‐specific diversity in protein interactions within stress granules. Cell 172, 590–604. 10.1016/j.cell.2017.12.032 PMC5969999 29373831

[30] Mehringer J , Do T‐M , Touraud D , Hohenschutz M , Khoshsima A , Horinek D and Kunz W (2021) Hofmeister versus Neuberg: is ATP really a biological hydrotrope? Cell Rep Phys Sci 2, 100343.

[31] Buchan JR and Parker R (2009) Eukaryotic stress granules: the ins and outs of translation. Mol Cell 36, 932–941. 10.1016/j.molcel.2009.11.020 PMC2813218 20064460

[32] Ju J‐S , Fuentealba RA , Miller SE , Jackson E , Piwnica‐Worms D , Baloh RH and Weihl CC (2009) Valosin‐containing protein (VCP) is required for autophagy and is disrupted in VCP disease. J Cell Biol 187, 875–888. 10.1083/jcb.200908115 PMC2806317 20008565

[33] Saha S , Weber CA , Nousch M , Adame‐Arana O , Hoege C , Hein MY , Osborne‐Nishimura E , Mahamid J , Jahnel M , Jawerth L *et al*. (2016) Polar positioning of phase‐separated liquid compartments in cells regulated by an mRNA competition mechanism. Cell 166, 1572–1584. 10.1016/j.cell.2016.08.006 PMC5034880 27594427

[34] DeLaBarre B and Brunger AT (2005) Nucleotide dependent motion and mechanism of action of p97/VCP. J Mol Biol 347, 437–452. 10.1016/j.jmb.2005.01.060 15740751

[35] Zhou HJ , Wang J , Yao B , Wong S , Djakovic S , Kumar B , Rice J , Valle E , Soriano F , Menon MK *et al*. (2015) Discovery of a first‐in‐class, potent, selective, and orally bioavailable inhibitor of the p97 AAA ATPase (CB‐5083). J Med Chem 58, 9480–9497. 10.1021/acs.jmedchem.5b01346 26565666

[36] Barkley RJR , Crowley JC , Brodrick AJ , Zipfel WR and Parker JSL (2024) Fluorescent protein tags affect the condensation properties of a phase‐separating viral protein. Mol Biol Cell 35, ar100. 10.1091/mbc.E24-01-0013 PMC11244164 38809580

[37] Pandey NK , Varkey J , Ajayan A , George G , Chen J and Langen R (2024) Fluorescent protein tagging promotes phase separation and alters the aggregation pathway of huntingtin exon‐1. J Biol Chem 300, 105585. 10.1016/j.jbc.2023.105585 PMC10825056 38141760

[38] Meyer H and Weihl CC (2014) The VCP/p97 system at a glance: connecting cellular function to disease pathogenesis. J Cell Sci 127, 3877–3883. 10.1242/jcs.093831 PMC4163641 25146396

[39] Bodnar NO , Kim KH , Ji Z , Wales TE , Svetlov V , Nudler E , Engen JR , Walz T and Rapoport TA (2018) Structure of the Cdc48 ATPase with its ubiquitin‐binding cofactor Ufd1‐Npl4. Nat Struct Mol Biol 25, 616–622. 10.1038/s41594-018-0085-x PMC6044470 29967539

[40] Bruderer RM , Brasseur C and Meyer HH (2004) The AAA ATPase p97/VCP interacts with its alternative co‐factors, Ufd1‐Npl4 and p47, through a common bipartite binding mechanism. J Biol Chem 279, 49609–49616. 10.1074/jbc.M408695200 15371428

[41] Jaffray EG , Tatham MH , Mojsa B , Liczmanska M , Rojas‐Fernandez A , Yin Y , Ball G and Hay RT (2023) The p97/VCP segregase is essential for arsenic‐induced degradation of PML and PML‐RARA. J Cell Biol 222, e202201027. 10.1083/jcb.202201027 PMC10005898 36880596

[42] Ritson GP , Custer SK , Freibaum BD , Guinto JB , Geffel D , Moore J , Tang W , Winton MJ , Neumann M , Trojanowski JQ *et al*. (2010) TDP‐43 mediates degeneration in a novel drosophila model of disease caused by mutations in VCP/p97. J Neurosci 30, 7729–7739. 10.1523/JNEUROSCI.5894-09.2010 PMC2890254 20519548

