# Supplementary material for: Valosin‐containing protein counteracts ATP‐driven dissolution of FUS condensates through its ATPase activity in vitro
Source: FEBS Lett. 2026 Mar 16;600(18):2787–99. doi: 10.1002/1873-3468.70328 (PMC13618296; doi:10.1002/1873-3468.70328)
Supplement: Supplementary file 1 [file FEB2-600-2787-s001.pdf]

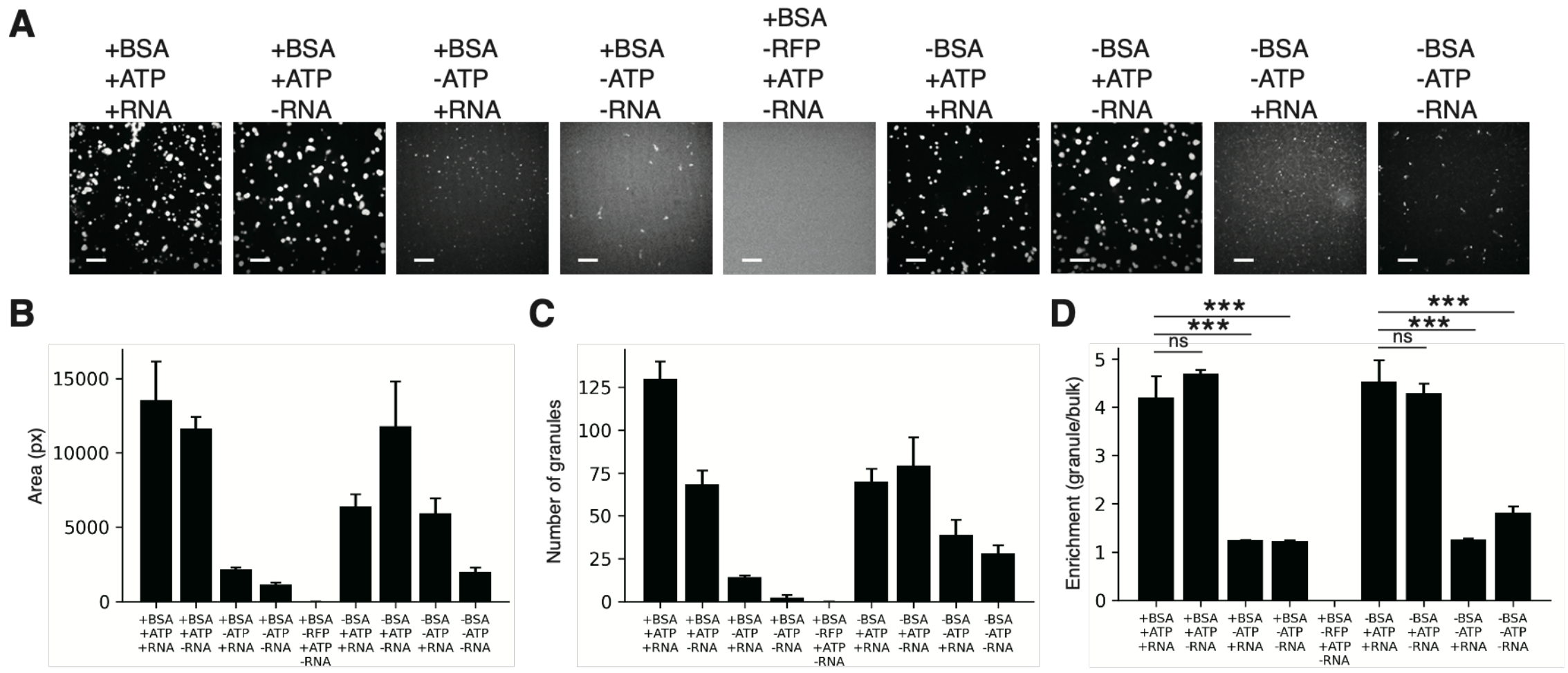

Supplementary Fig. S1. Control assays for LLPS components. (A) Representative images of RFP/BSA controls under various combinations of ATP and RNA. (B-D) Quantification of condensate parameters for controls, confirming that FUS LLPS is RNA-dependent and sensitive to ATP in the absence of VCP. Notably, RFP itself shows some condensate formation and relative stability to ATP, suggesting potential background effects from the His-tag or other non-specific interactions in the reconstituted system. Scale bars: 10 μm. Data represent mean ± SEM from n = 3 independent experiments (10 images per experiments). Statistical comparisons were performed between +ATP and −ATP conditions within each BSA/RNA group. Details are listed in Supplementary Table S1. ***$p < 0.001$, ns: not significant (Mann-Whitney U test).

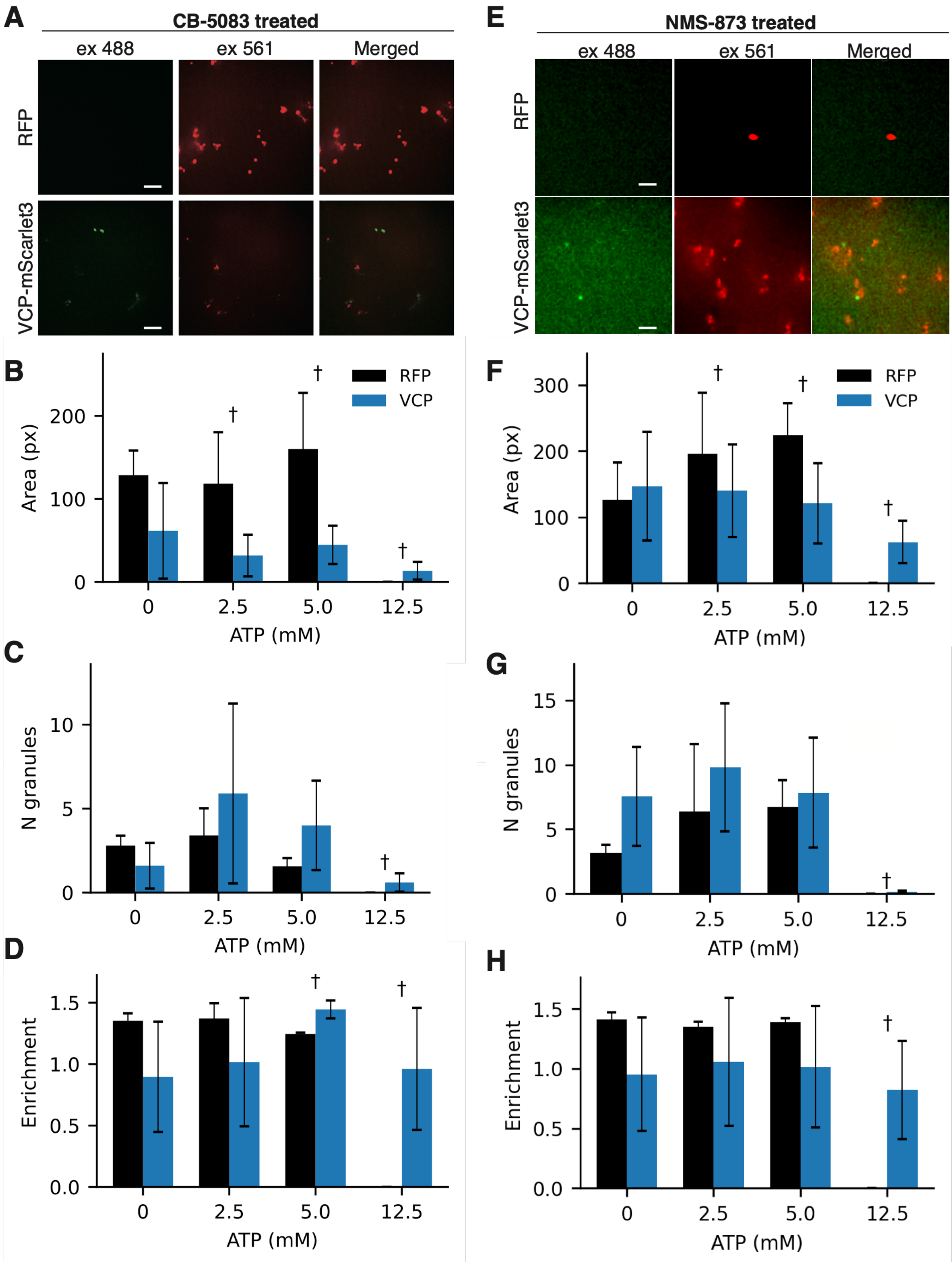

Supplementary Fig. S2. CB-5083 and NMS-873 differentially affect VCP-mediated condensate protection. (A-D) CB-5083 treatment. (A) Representative images of FUS condensates (WT) with RFP or VCP in the presence of CB-5083 at 12.5 mM ATP (60 min). (B-D) Quantification of condensate area (B), number (C), and enrichment (D). CB-

5083 did not abolish VCP's protective effect. (E-H) NMS-873 treatment. (E) Representative images with NMS-873. (F-H) Quantification showing NMS-873 abolishes VCP's protective effect, consistent with ML240 results. Scale bars: 10 µm. Data represent mean ± SEM from n = 3 independent experiments (10 images per experiments). † large effect size (|Cliff's δ| ≥ 0.474; Mann-Whitney U test). Statistical details are provided in Supplementary Table S1.

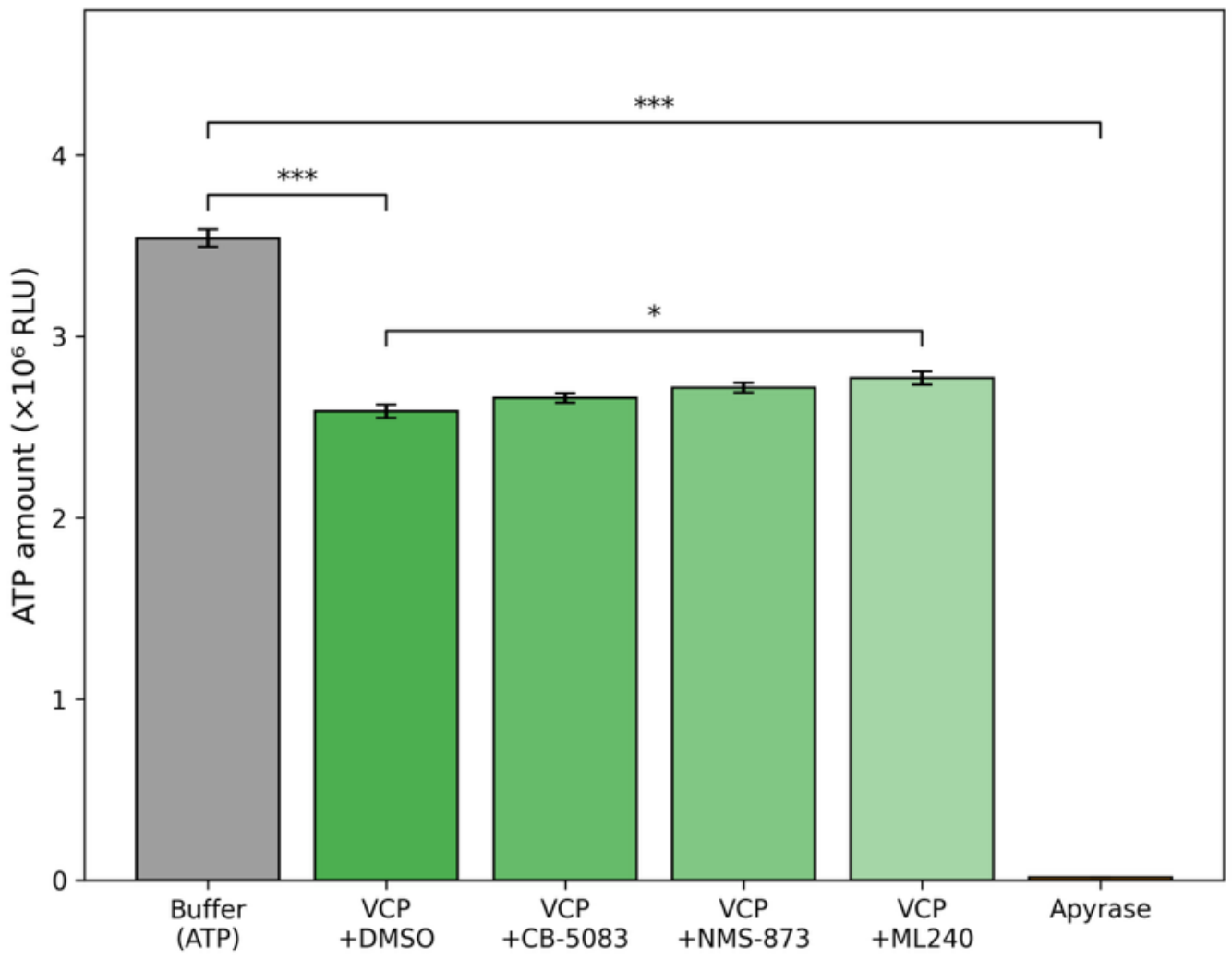

Supplementary Fig. S3. Direct measurement of ATP consumption by VCP. ATP levels were measured using CellTiter-Glo 2.0 luminescence assay after incubation with VCP and inhibitors. VCP significantly reduced ATP levels compared to buffer control (−27%, $p < 0.001$). ML240 treatment partially restored ATP levels (+7.1%, $p = 0.025$), while CB-5083 and NMS-873 showed no significant effect. Apyrase served as a positive control for ATP depletion (>99% reduction). Data represent mean ± SEM ($n = 3$ independent experiments). *$p < 0.05$, ***$p < 0.001$ (Welch's t-test). Statistical details are provided in Supplementary Table S1.
